# P-593. The Potential of Microbial Cell-Free DNA Metagenomic Sequencing to Uncover Public Health Gaps in Transmissible Infectious Diseases

**DOI:** 10.1093/ofid/ofaf695.807

**Published:** 2026-01-11

**Authors:** Sarah Y Park, Shivkumar Venkatasubrahmanyam, Eliza J Chang, Frederick S Nolte

**Affiliations:** Karius, Inc., Redwood City, California; Karius, Inc., Redwood City, California; Karius, Redwood City, California; Karius, Inc., Redwood City, California

## Abstract

**Background:**

Rapid identification of transmissible pathogens is critical to recognize public health threats. However, data for non-reportable pathogens are often lacking. In 2024 on infection listservs, clinicians reported concerns about acutely increasing parvovirus B19 (B19V) and *Mycoplasma pneumoniae* (Mp) infections, respectively. We describe reports of B19V and Mp by Karius Spectrum™, a validated, pathogen-agnostic plasma microbial cell-free DNA (mcfDNA) sequencing test used in hospital settings.

**Figure 1.**
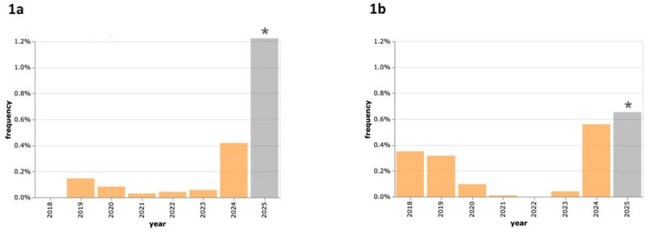
Plasma microbial cell-free DNA detections of parvovirus B19V (a) and Mycoplasma pneumoniae (b) among all patients tested, 2018–2025. Frequencies for 2025 (shown in gray, marked with an asterisk) subject to change, as data only includes the first two months.

**Methods:**

We analyzed B19V and Mp, detected and quantified in US patients’ plasma samples submitted to the Karius CLIA certified/CAP accredited laboratory from Apr 2018–Feb 2025. We characterized these detections based on result and test requisition form data and analyzed the data for any trends.

**Results:**

Of 85,977 patients tested during the study period, 47,183 patients yielded 49,508 positive samples from 49 states and the District of Columbia. B19V was detected in 211 (0.25%) patients (580 MPM median, IQR 111–6,708) from 33 states, and Mp in 218 (0.25%) patients (407 MPM median, IQR 106–1,408) from 27 states. B19V detections ranged from 0.03–0.15% in 2019–2023 (no B19V detections in 2018), with decreased detections during 2021–2022. There were increased Mp detections in 2018 (0.28%) and 2019 (0.33%), which then decreased in 2020–2023 (0–0.11%). Detections of both pathogens increased in late 2023 and continued through 2024, with B19V detected among 0.42% and Mp among 0.55% of tested patients in 2024 (one-sided Z-Score p-value 0.01 and 0.03, respectively; Figure 1). 2024 detection increases were proportionately greater among pediatric than adult patients for both B19V (z-score difference of 0.11) and Mp (0.49).

**Conclusion:**

Plasma mcfDNA sequencing data confirmed increased activity of non-reportable transmissible pathogens noted in listservs and may contribute to overall situational awareness of such pathogens. Detections may be used to track impacted populations and even potentially identify specific genomic characteristics (https://doi.org/10.1093/infdis/jiad452). Establishing stronger relationships between public health and commercial laboratories may enhance public health surveillance and responses to emerging threats.

**Disclosures:**

Sarah Y. Park, MD, FAAP, Karius, Inc.: current employee Shivkumar Venkatasubrahmanyam, PhD, Karius, Inc.: current employee Eliza J. Chang, A.B., Karius, Inc.: current employee Frederick S. Nolte, PhD, D(ABMM), F(AAM), Karius, Inc.: current employee

